# Processing of social exclusion in a strict hierarchy

**DOI:** 10.1371/journal.pone.0338212

**Published:** 2025-12-19

**Authors:** Michael Niedeggen, Rudolf Kerschreiter

**Affiliations:** 1 Division of Experimental Psychology and Neuropsychology, Department of Education and Psychology, Freie Universität Berlin, Berlin, Germany; 2 Division of Social, Organizational, and Economic Psychology, Department of Education and Psychology, Freie Universität Berlin, Berlin, Germany; Ritsumeikan Asia Pacific University: Ritsumeikan Asia Taiheiyo Daigaku, JAPAN

## Abstract

Exclusion is defined as a significant reduction of involvement or interaction with others. This threatens our social need for belonging and triggers a negative affective response. The response is associated with a P3 effect in the event-related brain potentials (ERP) indicating that the processing of exclusion is characterized by a violation of the expected social involvement. The majority of experimental findings relies on the *Cyberball* setup, in which two putative co-players exclude the participant in a virtual ball-tossing game. The current studies challenge the previous findings by introducing a novel paradigm, *Cyberband*, which simulates participation in an orchestra setting. Here, exclusion is defined by reducing the number of cues provided by a putative conductor. In experiment 1, the number of cues exclusively directed to the individual (solo) was reduced. In contrast to previous *Cyberball* findings, the self-reported threat to belonging as well as the P3 effect were clearly diminished. In experiment 2, participants were excluded from joint actions. Here, a reduced participation in common actions (tutti), but not in dyadic actions (duets), enhanced the P3 effect. In both experiments, the reduced participation primarily affected the expression of an early frontal ERP positivity (P2) indicating a change of cue salience. In sum, these results indicate that exclusion is processed differently if attributed to a single decider: The experience is reported to be less painful, and ERP signatures of a violation of the expected participation – prominent in the established Cyberball setup – are predominantly elicited by an exclusion from joint actions.

## Introduction

Social interaction and belonging are fundamental social needs that are essential for our feeling of security [1]. Sustained social rejection leads to physical and psychological distress that might affect mental health and well-being [2].

In the following, we will refer to this process as social exclusion, which embraces the intentional and non-intentional exclusion from social interaction and contribution [3]. According to the seminal temporal need-threat model of Williams [4], the slightest hint of exclusion will be detected by a pre-attentive system and induces an aversive neural response that shares the characteristics of physical pain [5]. This process motivates a palliative response – such as the attempt to reconnect – and to reduce the psychological discomfort [6].

**Fig 1:**
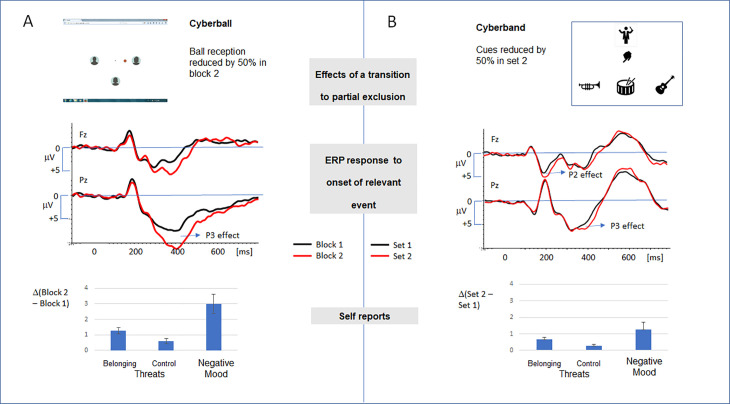
A comparison of the Cyberball and Cyberband setup and the effects of a partial exclusion. **(A)** Cyberball is a virtual ball-tossing game. If the participant is excluded by the putative co-players, threat to the social need ‘belonging’ is elicited accompanied by an increase in negative mood. In the ERP responses – time-locked to the relevant event “ball reception of the participant” – the transition to an exclusion (black line: 33% ball reception vs. red line: 16% ball reception) elicits a marked P3 effect. The data refer to the results of a previously published study [11]. **(B)** In the Cyberband setup, the participant is assigned to an instrument, and a response is required if the conductor provides a cue. A selective reduction of the number of cues induces a modest threat to belonging, and a negligible increase in negative mood. As for ERPs, the onset of the relevant event was defined as the onset of the cue requiring action of the participant. When reducing the cue probability between set 1 (black line: 33%) and set 2 (red line: 16%), a marked frontal P2 effect and a moderate parietal P3 effect is elicited (see text). The ERP traces and self-report values refer to the effects summarized for both experiments (n = 67). Error bars refer to the standard error of mean (SEM).

The physiological and psychological processes associated with the processing of exclusionary events have been examined in different experimental setups – inspired by real-life situations: This included a neglect in a chat room [7], web conference [8], or at a lunchroom Table [9]. The most established task is the Cyberball game [10]: According to the cover story, participants are instructed to imagine playing a ball-tossing game. The two putative co-players – depicted as avatars on a computer screen (see Fig 1a) – are actually computer-generated. Following a series of ball throws in which the participant is included, the participant will be neglected as the game continues. In other words, the co-players will exclusively pass the ball to each other.

Research in the past two decades indicated that the processing of exclusionary events in the Cyberball paradigm is associated with a stable response in the self-reports and in the event-related brain potentials (ERPs): As for the self-reports, a tailor-made questionnaire (need-threat questionnaire, NTQ, [4]) has been developed: The corresponding scale ‘belonging’ indicates reliably if an individual felt excluded in the Cyberball game [12]. The exemplary results depicted in Fig 1a refer to a previous study see: [11]), and already signal that the effect is usually not restricted to a threat to the need of belonging, but spreads to the other scales included in the NTQ, namely the need for ‘self-esteem’, ‘meaningful existence’, or ‘control’. In line with the predictions of the temporal need-threat model, being excluded also significantly enhances negative mood. The latter response is in line with the assumption of an overarching ‘inconsistency compensation approach’ [6]: According to this model, the experience of exclusion in the Cyberball game is not in line with our subjective beliefs and expectations on the upcoming social interaction, and therefore defines an aversive event.

Notably, this account is supported by numerous ERP studies: Summarizing the results of previous research, a recent meta-analysis revealed that the processing of exclusionary signals is reliably associated with an increase of the P3 component [13]. This *P3 effect* is reliably elicited if an inclusionary experience is followed by an exclusionary experience [11]. The increase of the P3 amplitude associated with this transition (see Fig 1a) is not due to the reduction of the relevant event, but to the violation of the a priori expectations on social participation. E.g., the P3 effect is not expressed if the transition to an exclusionary setting is associated with an increase in the number of co-players [14]. Further evidence is provided by studies indicating that the expression of the P3 effect critically depends on previous experience [11] or the self-assigned social power [15,16]: If experimental manipulation suggests an increase in social power, the P3 effect elicited by a transition to exclusion is increased. These research lines suggest that a bias in the a priori expectation of expected social involvement affects the processing of exclusion in the Cyberball scenario. This idea was additionally supported in clinical studies [17], indicating that a changed sensitivity for exclusionary signals, such as in Borderline-Personality Disorder, is also associated with a corresponding change of the P3 effect. In sum, the ERP research in the last decades – primarily using the Cyberball paradigm – indicates that the processing of exclusionary cues reliably triggers a component related to the state of subjective expectancy. Notably, the effect can hardly be explained by the activation of a pre-attentive alarm system, as assumed in the temporal need-threat model. According to the need-threat model, an early invariant response would be expected.

Despite its reliability, it is questionable whether the Cyberball setup completely covers all relevant psychological processes related to social exclusion. One important difference between cyberball and social participation in education or work life concerns the exclusionary agent: while the exclusionary signal in cyberball stems from an equal partner, in education or work settings, exclusion or inclusion often relies on the decision of a superordinate agent. A second difference is the sequential action sequence in Cyberball, which neglects the joint interaction of several individuals. To overcome these limitations, we introduce a novel experimental setup, further labeled as *Cyberband*, which allows us to examine the impact of these factors and to address the corresponding research questions.

The first question relates to the source of the exclusionary action: in the Cyberball setup, being neglected can be attributed to the coplayers’ decision. Importantly, experimental findings suggest that the attribution of an intention is probably not even necessary: In a series of studies, Zadro and colleagues [18] showed that the feeling of exclusion is also elicited if the participants are informed of the scripted ‘behavior’ of the co-players. Moreover, the credibility of the cover story has no significant impact on the effects elicited by exclusion: For example, the physical presence of the (putative) co-players in the lab increased the credibility, but does not affect the expression of the self-reported threat, or the expression of the P3 effect [19]. In sum, experimental evidence suggests that the immediate response to exclusion is less affected by attribution processes than by expectation.

The second question relates to an inherent limitation of the Cyberball setup: The ball throwing game implies a single recipient, i.e., the event of ball reception is not ‘shared’ by the co-players. However, in team sports or work organizations, prompts for joint actions are also essential. More importantly, the prompts for joint action imply a co-representation of the co-players’ action [20,21]. Previous studies suggest that this co-representation can be modulated by a preceding experience of exclusion [22,23]. In turn, these findings suggest that the markers of processing exclusionary signals (e.g., the P3 effect) will be modulated if prompts for joint actions are presented.

The new Cyberband setup (see Fig 1b) allows us to address both questions: Simulating a music band, the participant assigns themselves to one of three instruments, while the remaining instruments are played by putative co-players. The participant’s task is to press a response button if signaled by a corresponding cue for their instrument from the conductor. If the cue addresses the instruments of the co-players, the response must be withheld.

This setup parallels the Cyberball setup in several respects: Both simulate a social interaction with a limited number of co-players. Moreover, clear prompts are provided that require a response of the participant, and the probability of these self-relevant target events can be varied. In contrast to the Cyberball, in Cyberband the probability for involvement is defined by a single, superordinate agent (conductor). Moreover, a prompt might be relevant for one player (further labelled as single action), or it might the relevant for two (or more) players (further labelled as joint action).

In the present studies, we tested whether a reduction in self-relevant prompts in the Cyberband paradigm elicits processes comparable to a reduction in ball reception in the Cyberball paradigm, namely a self-reported threat to social needs as reflected in the standardized questionnaire (NTQ), and the established P3 effect. In the first experiment, we examined the effects of a reduction of self-relevant events in the Cyberband setup. The experimental design is based on the within-subject design established for the Cyberball [11,14–16]: In the first set, the number of prompts (further labeled as *cue*) was equally distributed across the different instruments (inclusion). In the second set, the number of cues was reduced for the participant’s instrument and enhanced for the other instruments (partial exclusion).

Based on the experimental effects elicited by a partial exclusion in the Cyberball setup, we expected to observe the following effects on the level of ERPs and self-reports:

For the ERPs, we hypothesized that the P3 response to cues will increase in set 2 as compared to set 1 (P3 effect). Based on a previously supposed expectancy-violation model, this increase is supposed to signal that a deviance to the expected probability to get involved in the game is detected [24].

For the self-reports, we hypothesized that the reduction of cues will elicit a threat to the social need ‘belonging’ – which serves as the most-stable indicator for exclusion in the Cyberball paradigm [12]. Since a threat to belonging is an aversive experience commonly associated with negative affect [11], we expected a corresponding increase in negative mood.

## Methods: Experiment 1

For experiment 1 and 2, the procedures were approved by the Ethics Committee of the Department of Education and Psychology, FU Berlin (No.026.2023, November 1, 2023) and written informed consent was taken in all participants according to the Declaration of Helsinki. All data (self-reports, pre-processed EEG data) are available in a data repository, as well as the program code. All measures and effects of the experimental manipulations are reported, and criteria for exclusion of data sets are detailed below.

### Participants

The *a priori* calculation of the sample size was focused in the detection of a P3 effect elicited by the transition-to-partial exclusion. Based on the results of a previous study [11], we assumed that the P3 effect is reliable expressed and will result in a mid-to-high-sized effect (*f* = 0.325 adjusted to the taxonomy of Cohen) for the hypothesized within-factor “experimental block” (two sets). According to G*Power [25], a sample size of 22 participants was required to detect an effect with a power of 80% and an alpha at 0.05.

Based on previous Cyberball studies [26], a rejection rate of 20% was considered. Thus, a total sample of 29 participants (20 female, 7 male, 2 diverse, age range: 18–38 years) was recruited at the FU Berlin. Recruitment started on November 11, 2023, and ended February 10, 2024. Most participants were undergraduate students. EEG data were rigorously checked for artefacts (see: Data analysis), leaving a final sample comprising 23 participants (18 w, 4 male, 1 diverse, mean age: 23.74, SD: 5.34). Although the study did not include professional musicians, the effect of ‘expertise’ was controlled for in an a posteriori analysis. To this end, participants were asked whether they play an instrument and have experience playing in a band. This was confirmed in 12 out of 23 participants. To increase the power of this analysis, the data of study 2 were additionally considered (see point: Limitations).

### Task and design

Before the experiment started, participants were provided with a cover story on the aims of the study: They were informed that the orchestra simulation aims to test the effect of visual imagination in a vigilance task. Following this cover story, it is necessary to maintain a visual representation of the scenario, including of co-players – assigned to the other instruments – and the conductor. The vigilance will be monitored by the timely and accurate response to the cues, as well as by the EEG signals. As in the Cyberball task, the information on the upcoming exclusion was not provided a priori in order to prevent a bias in the subsequent self-reports. In contrast to the typical Cyberball design, participants were not told that they will interact with co-players physically present. In contrast, they were informed that co-players and the conductor are computer-generated. As demonstrated previously, the credibility of the cover story will not affect the expression of the effects in self-reports and ERPs [19].

**Fig 2:**
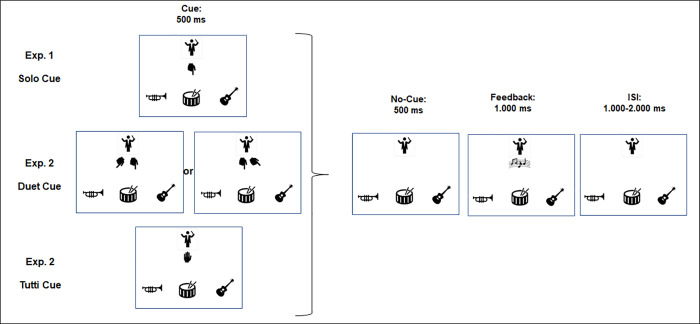
Definition of a trial in Cyberband. The visual cue was presented for 500 ms, and then vanished for another 500 ms. Relevant cues required a response (press the space bar of the keyboard) within a response window of 750 ms. The relevant cue directed to the participant’s instrument – changed in frequency in set 2 – was a solo cue in experiment 1. In experiment 2 it was either a duet (EG duet) and a tutti cue (EG tutti). The cue vanished for 500 ms. In the subsequent feedback period, a short auditory sample containing the instrument of the participant was played if the response was correct and in time. In case of a miss of a solo, only the picture of the notes appeared, and no sound sample of the participants instrument was played.

The experiment (see Fig 1 and Fig 2) was programmed in PsychoPy2 (version V1.85.6 [27]). First, the participant assigned themselves to one of three possible instruments (drum, guitar, or trumpet). Then, the visual setup was displayed on the computer screen (7° x 7° at a viewing distance of 120 cm with the participant’s head stabilized by a chin rest): In the bottom row, three instruments were presented. The instrument selected by the participant was always placed in the center. Above the instruments, an icon represented the conductor. The participant was instructed to fixate on this icon.

The start of a trial (see Fig 2) was defined by the cue: The symbol of a hand was presented below the icon representing the conductor. In case of a *solo* cue, the index finger was directed to the corresponding instrument. The self-relevant event was defined by a cue addressing the instrument assigned to the participant. A second, less frequent, cue was defined by a *tutti* signal: Here, the palm of a hand with all fingers was presented, indicating that all instruments are requested to play. Each cue was presented for 500 ms and then vanished for 500 ms. In case of a relevant cue, the participant had to press the space bar of a keyboard during the visual presentation of the cue. Since pilot data revealed a noticeable miss rate, the response time window was extended for another 250 ms. 500 ms following the offset of the cue, the feedback period was announced by a visual cue (sheet music) for 1.000 ms. If the participants responded in time to a relevant solo cue, a sound sample (1,000 ms duration) of the instrument assigned to the participant was played. In case of a miss – or of a false alarm, the sample was not played. If the participants responded in time to a relevant *tutti* cue, a sound sample (1,000 ms duration) including all instruments was played. In case of a miss, the sound sample did not contain the participants’ instrument. If the cue did not address the participant’s instrument, no music sample was played. Following the feedback signal, an inter-stimulus-interval (ISI) varying between 1,000 and 2,000 ms preceded the onset of the next trial. Eye blinks were encouraged within this period.

In order to familiarize the participant with the task, each experiment began with 13 practice trials (four soli for each instrument and one tutti). This was followed by two experimental sets, each comprising 130 trials, and lasting approximately 12 minutes. The sets were interrupted by a short break of approximately three minutes. Supporting the cover story, in the break, participants were reminded to visualize the orchestra setting and to keep focused on the conductor providing the cues.

In set 1, the number of cues was equally distributed for the instruments: Each instrument received 40 cues. In addition, 10 *tutti* cues were interspersed to support the music band simulation. This resulted in a total of 130 cues. In set 2, the number of solo cues for the participant’s instrument was reduced by 50% (20 cues), whereas the cues for the other instruments were enhanced (50 cues per instrument). A corresponding reduction in Cyberball (partial exclusion [11,14]) was found to be sufficient to elicit reliable effects in self reports ERPs. The number of the *tutti* cues was not changed (10 trials), resulting in a total of 130 cues. Please note that event ‘solo cue for the participant’s instrument’ served as the probe for the expectancy state, and therefore corresponds to the event ‘ball reception’ in the Cyberball game. The number of cues in each set targeting the participants’ instrument (set 1: 40, set 2: 20) defines the number of events available for the ERP analysis. The experimental design is provided as a table in the supplement (S1 Table).

Following the second set, the participants completed the NTQ measuring the threat to four social needs (belonging, self-esteem, meaningful existence, and control, each scale based on three items), and a negative mood scale based on an eight-item adjective list (e.g., “I felt sad” or “I felt angry”, [28]). Both questionnaires are established self-reports in Cyberball studies and reflect the exclusionary status reliably [12]. To reduce the number of questionnaires and the memory load, self-reports were provided as a relative rating: Participants had to estimate the change in threat and mood, respectively, on a 7-point Likert scale (“-3” = much stronger in set 1 to “3” = much stronger in set 2). This procedure has been applied successfully in previous Cyberball studies [26].

The participants were debriefed following the completion of the questionnaire: They were informed on the primary aim of the experiment, namely to explore the processing of reduced involvement. Correspondingly, the written consent was asked for at the beginning and the end of the experiment. The completion of the experiment – including the electrode montage – required approximately 90 minutes.

### EEG recording

To allow a comparison of the ERP effects elicited by a transition-to-exclusion in the Cyberband and Cyberball design, the settings of the EEG recoding were parallelized to a previous study [11]: Ag/Ag/Cl electrodes were embedded in an elastic cap (EASYCAP, Herrsching, Germany; BrainAmps amplifier, BrainProducts, Gilching, Germany) of a size corresponding to the circumference of the participant’s head. The position of the eight active EEG electrodes approximated the following positions of the 10–20 system: AFz, Fz, F3, F4, Cz, Pz, P7, and P8. Signal were referenced to linked earlobes, with FCz serving as ground. For all EEG electrodes, impedance was kept below 10 kΩ.

Electrodes were connected to the amplifies (BrainAmps amplifier, BrainProducts, Gilching, Germany), and the signal was monitored running the software ‘BrainVision Recorder’ (Version: 2.1, Brain Products, Gilching, Germany). EEG data were recorded continuously and sampled at 500 Hz. The online signal was band-pass filtered (0.1–100 Hz) and the effect of AC hum was reduced by applying a notch filter (50 Hz). Ocular artifacts were controlled by recording vertical and horizontal electrooculograms (vEOG, hEOG).

For offline analysis, the software ‘Vision Analyzer’ (Version: 2.1, Brain Products, Gilching, Germany) was used. The following protocol was also applied for the analysis of EEG data in the Cyberball experiments (see Fig 1a, [11]) which serve as a landmark: First, data were bandpass filtering (0.3 to 30 Hz, 24 dB/Oct). Second, EEG signals were epoched (−100 ms to 800 ms) separately for the sets. Analysis was focused in the events “*solo* cue to the participants instrument”. Here, the P3 effect was hypothesized which should reflect a systematic change in the state of expectancy [16]. The responses to the event “*solo* cues to other instrument” were analyzed additionally in an *a posteriori* analysis. Third, the EEG and EOG channels were baseline-corrected (−100–0 ms). Fourth, a semi-automatic artefact control procedure was applied: EOG channels were automatically controlled for ocular artifacts (EOG > 100 μV), and affected trials were rejected from the analysis. Then, EEG trials were first marked if an amplitude threshold was exceeded (EEG > 75 μV), and rejected if this was due to high alpha activity or movement artifacts. In a final manual correction, EEG trials were analyzed for slow linear drifts affecting the baseline period, or high-frequency bursts. Following this rigorous artifact rejection, trials were averaged: Averages for each participant were separated for the different types of cues, electrodes and the two sets of the Cyberband game.

In accordance with our previous Cyberball studies, we discarded the data of a participant from analysis if the averaged ERP response to the event of interest (*solo* cue) relied on less than 14 trials. In set 1, the mean number of trials included in analysis was 31.6 (SD: 4.42) reflecting a trial rejection rate of 21%. In set 2, the mean number of trials included in analysis was 16.0 (SD: 2.73) reflecting a trial rejection rate of 20%.

### Data analysis

#### Behavioral and questionnaire data.

For each participant, a difference value (relative judgement: set 1 – set 2) for each NTQ scales (belonging, self-esteem, meaningful existence, and control) and negative mood was available. The data were first analyzed descriptively. For the scales of interest (threat to belonging, negative mood), a one-sided t-test was computed. Furthermore, we explored whether other threats (self-esteem, meaningful existence, control) are affected by the experimental manipulation.

#### ERP data.

The determination of the time regions also considered the grand-averaged ERPs of the study 2 (*joint action* conditions) to allow a comparison of the results. To this end, the grand-averaged ERPs responses to the event ‘self-relevant cue’ – reduced in the second set – were computed. For the resulting ERPs, the global field power (GFP) index was determined, and time windows with marked differences were explored. The GFP shows a maximum at 380 ms. Based on the previous Cyberball studies, we expected differences between the sets in a P3 region extending from 320 to 420 ms [29,30]: To account for the length of the time windows used in previous Cyberball studies (80 ms), as temporal window in the time range 340–420 ms was defined. Mean amplitudes for this time window were computed for the ERP data of each participant separately for the experimental condition ‘set’ and electrodes.

Marked differences between set 1 and set 2 were also observed in the expression of a more-transient positivity starting at 200 ms and peaking about 220ms. Since the topography revealed that the differences between were mostly expressed at fronto-central leads, the corresponding effects apparently refers to a P2 component also observed in Cyberball studies [19,31,32]. In an exploratory analysis this effect, we extracted the mean amplitude – defined in the time range 200–240 ms – separately for the electrodes and conditions. Please note, that the transient character of the P2 also allows a peak identification, so that the P2 amplitude and latency were determined (procedure ‘peak detection’, Brain Vision Analyzer 2.0). To increase the reliability of the peak detection, the procedure was applied to the pooled ERP response at the frontal electrodes (AFz, Fz, F3 and F4). Results of the peak amplitude and mean amplitude analysis corresponded (see S2 Table).

As for the P3 segment, a 2 x 4 ANOVA was conducted comprising the repeated-measure factors ‘set (1 vs 2) and electrode position (Cz, Pz, P7, P8). The choice of the centro-parietal electrode cluster is not only in line with the topography observed in this study, but also with the cluster defined in previous Cyberball studies [11]. For the P2 amplitude and latency, a corresponding analysis was performed. The topography clearly indicated a frontal expression of the P2 effect – already observed in previous Cyberball studies [31] – and correspondingly a frontal electrode leads (AFz, Fz, F3, F4) were considered. The results will report the Greenhouse-Geisser corrected p-values. In case of a significant interaction, post-hoc comparisons were conducted. SPSS (version 27, IBM) was used for the statistical analysis.

As mentioned above, analysis did also include the responses to the event “*solo* cue for other instrument”. The processing of the cues exclusively directed to the co-players did not show a systematic difference between the sets (supplement S2 Figure). Grand-averaged data for the events “tutti cue” are provided in the supplement (see S1 Figure). Please note that a statistical analysis for this event was not possible due to the limited number of artefact-free trials (set 1: M = 9.1 trials, SD = 0.76; set 2: M = 9.7 trials, SD = .65).

## Results: Experiment 1

### Self-reports and reaction times

Based on our hypothesis, the analysis was focused on the expression of the threat to belonging and negative mood (see Table 1).

**Table 1:** Self-reports effects and response times in experiment 1 and 2.

	Experiment 1 *Solo*	Experiment 2 EG: *Duet*	Experiment 2 EG: *Tutti*
NTQ: Belonging (ΔSet 2 – Set 1)	.52 (.232) .06,.98	.92 (.237) .45, 1.40	.55 (.237) .07, 1.02
NTQ: Meaningful Existence (ΔSet 2 – Set 1)	.55, (.227) .10, 1.01	.91 (.232) .45, 1.37	.58 (.232) .11, 1.04
NTQ: Self Esteem (ΔSet 2 – Set 1)	.23, (.239) −.25,.71	.70 (.245) .21, 1.19	.71 (.245) −.22, 1.20
NTQ: Control (ΔSet 2 – Set 1)	−.16, (.172) −.50,.18	.21 (.176) −.14,.56	.76 (.176) .41, 1.11
Negative Mood (ΔSet 2 – Set 1)	.87 (.74) −.61, 2.35	1.80 (.76) .28, 3.31	1.14 (.76) −.38, 2.35
Response Time (ms) Set 1	653.7 (10.5) 633.0, 674.3	662.3 (10.6) 641.2, 683.3	632.7 (10.6) 611.1, 653.3
Response Time (ms) Set 2	641.1 (12.5) 616.2, 666.0	646.9 (12.7) 621.4, 672.3	635.8 (12.7) 610.3, 661.2

Caption: The questionnaire data rely on a relative rating of individuals, and positive values indicate that the effect was stronger in set 2 as compared to set 1. In each cell, the first line refers to the mean value and the standard error of mean, and the second line to the lower and upper limits of the confidence interval (95%).

Compared to the first set, participants reported a modest increase in the threat-to-belonging, and the one-sided one-sample t-test signaled a significant increase, *T(22)=2.11, p = 0.023, d = 0.439*. Negative mood was only slightly enhanced by a transition-to-exclusion, and the effect was not statistically significant, *T(22)=0.94, p = 0.179, d = 0.196*.

From the remaining scales (see Table 1), only ‘Meaningful Existence’ responded to the transition-to-exclusion, which was significantly expressed in a one-sided comparison, *T(22)=2.06, p = 0.026, d = 0.430*. No effects were obtained in the scales ‘self-esteem’ and ‘control’.

Since a speeded response was required in the Cyberband setting, the response times were analyzed additionally (see Table 1). The descriptive statistics indicated that the mean response time was slightly reduced in the second set, but this difference was not statistically significant, *F(1,22)=2.87, p = .104, η*^*2*^_*p*_* = .116*.

### ERP effects: Effects of cue reduction

**Fig 3:**
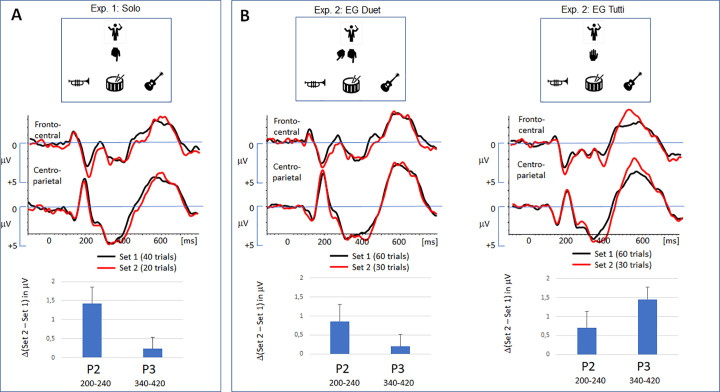
Grand-averaged ERPs for study 1 (solo cue) and study 2 (duet and tutti cues). The ERP traces refer to the response to the relevant cue in set 1 (black) and set 2 (red). The bar graphs represent the mean amplitude of the ERP effect (set 2 – set 1) in the time range of the P2 and the P3. Error bar refer to the SEM. **(A)** Effects in experiment 1. The reduction of the solo cue enhanced the expression of a frontal positivity at 200 ms (P2). The effect was less expressed in the time range of the P3. **(B)** Effects for duet reduction in experiment 2: The P2 and P3 effect, observed in the solo group **(A)**, can be replicated in the corresponding time ranges. **(C)** Effects for tutti reduction in experiment 2: As compared to the solo group, the frontal P2 effect is slightly reduced. Notably, the P3 effect is clearly enhanced.

As for the ERPs, analysis was focused on the events (here: cue onset) which differed with respect to frequency between the two sets (Number of solo cues: 40 in set 1 vs 20 in set 2). As stated above, a corresponding reduction was defined in Cyberball studies as a ‘transition to partial exclusion`. The effects of the reduction of *solo* cues are depicted in Fig 3a. A marked posterior negativity peaking at about 180 ms is followed by a frontal positivity (P2) at about 200 ms. At 300 ms, a sustained positive shift mostly expressed at centro-parietal leads can be observed. The positivity is mostly expressed in the time range 350–380ms, and returned to baseline level at 500 ms. The corresponding descriptive data are provided in Table 2.

**Table 2:** ERPs effects of cue reduction (set 1 vs set 2) in experiment 1 and 2.

	Experiment 1 Solo		Experiment 2 EG Duet		Experiment 2 EG Tutti	
	Set 1	Set 2	Set 1	Set 2	Set 1	Set 2
P2 (200–240 ms)	2.29 (.43) 1.43, 3.14	3.71 (.51) 2.70, 4.72	1.52 (.44) 0.65, 2.39	2.38 (.52) 1.35, 3.41	1.74 (.44) 0.87, 2.61	2.44 (.52) 1.41, 3.47
P3 (340–420 ms)	3.91 (.55) 2.81, 5.01	4.14 (.52) 3.11, 5.18	3.66 (.56) 2.54, 4.78	3.86 (.53) 2.80, 4.92	2.56 (.56) 1.44, 3.69	4.01 (.53) 2.96, 5.07

*Caption: For each, the P2 and the P3, the mean amplitudes in the corresponding time ranges (200–240ms, 340–420ms) are provided. In each cell, the first line refers to the mean value and the standard error of mean, and the second line to the lower and upper limits of the confidence interval (95%).*

Our research hypotheses addressed the time range of the P3 extending from 340 to 420 ms. Here, the most reliable effects of exclusion were observed in Cyberball studies. In the Cyberband setup, a clear increase in the amplitude of the P3 in set 2 was not expressed: In the selected P3 range (340–420 ms), amplitudes were at comparable level in the centro-parietal cluster, *F(1,22)=.40, p = .532, η*^*2*^_*p*_* = .018*, and the lack of an interaction with ‘electrode position’ indicated a homogenous cluster, *F(3,66)=2.23, p = .116, η*^*2*^_*p*_* = .092*.

More pronounced than the late P3 effect was a frontal P2 effect (see Fig 3A): In the second set, the P2 amplitude in the selected time range (200–240 ms) was clearly enhanced, *F(1,22)=7.65, p = .011, η*^*2*^_*p*_* = .258*. The effect was not modulated by electrode position, *F(3,66)=0.58, p = .584, η*^*2*^_*p*_* = .026*. Please note that the effect was confirmed for the P2 peak amplitude, *F(1,22)=19.24, p < .001, η*^*2*^_*p*_* = .267* (see S2 Table).

Furthermore, the descriptive analysis of the control event *tutti* (see supplement: S1 Fig) suggests that a P3 effect: Descriptively, an increase in amplitude was observed in set 2. The analysis remained descriptive because the number of trials was limited (n = 10).

## Discussion: Experiment 1

The findings of experiment 1 can be summarized as follows: The reduction of self-relevant events in the Cyberband design elicited a slight enhancement in the threat to the need for belonging, but did not induce a significant increase in negative mood. In the P3 range, the transition to exclusion induces a slight enhancement in amplitude, which was not significantly expressed. Instead, a significant increase of the frontal P2 was observed.

As for self-reports, the reduced size of the effects is notable. In the Cyberband setup, the effect sizes on the scales “threat to belonging” (Cohen’s d = 0.424) and negative mood (Cohen’s d = 0.196) are rather small. Using the same rating procedure in a Cyberball setup [11], the threat to belonging induced by reduced ball reception probability (50% reduction) was strongly expressed (Cohen’s d = 1.278), as well as the increase in negative mood (Cohen’s d = 1.026). This difference extends to the expression of the P3 effect: In the Cyberball study, the P3 amplitude increase induced by a transition-to-exclusion was clearly expressed (η^2^_p_ = .557) [11]. In the Cyberband setup, the effect size was substantially reduced (η^2^_p_ = .018).

Three factors might account for the differences in the processing of exclusionary events in the current design: First, in contrast to Cyberball, the Cyberband setup does not use a deception concerning the existence of the co-players. Yet, this slight change in the cover story can hardly explain the differential response pattern: Based on previous Cyberball studies, the credibility of the cover story does not affect the self-reported threats [18], nor does it modulate the expression of the P3 effect [19]. Second, Cyberband requires a differentiation of visual cues and a speeded response. Although additional cognitive effort might modulate the expression of the P3 amplitude [33], one has to consider that the effect can also be observed in complex tasks [34]. Third, the Cyberband setup introduces a conductor. This salient characteristic resembles the introduction of a putative supervisor in the ‘intervention Cyberball’ [35]: This supervisor might interfere with the decision of the participant on the recipient of the ball throw. The intervention of the supervisor elicited a reliable threat to social needs (here: control) and a clear P3 effect [26]. However, the ‘conductor’ in the Cyberband experiment is rather a distributor of goods (or here: cues), comparable to the setup of the dictator game [36]. If the introduction of the superordinate agent in a hierarchical setup is responsible for the lack of the P3 effect and the reduced sensitivity of participants in self-reports, we have to consider the possibility that the expectancy violation process is markedly reduced – or not activated at all.

A reduction of the degree of expectancy violation might be due to the setting of a priori expectations: Although participants might expect pro-social behavior also from a superordinate agent [37], a clear expectation of the specific social share can hardly be stated a priori. If expectations on the participation are not established, a re-adjustment of the expected involvement is less effortful. As predicted by the theoretical framework of an expectancy violation account [38,39], a rapid adaptation of expectancy level might be signaled by the modest P3 effect. Moreover, the neglect by the conductor is not as aversive as the neglect by co-players, and might therefore not trigger an aversive response [5].

Alternatively, one might assume that an expectancy violation process is not activated by default in a hierarchical setup. This idea is partially supported by previous behavioral studies indicating that the acceptance of social norms reduces the affective response to exclusionary signals in the Cyberball paradigm [40]. In experiment 2, we will follow up on both accounts.

In contrast to the hypothesized P3 effect, a frontal P2 effect was clearly expressed. Previous studies suggest that an increase of this component might be related and to the detection of salient events [41,42]. Notably, a P2 effect has also been observed in previous Cyberball studies: It can be reliably evoked by a transition-to-overinclusion, i.e., the participant receives the ball more frequently than the co-players [31,32,43]. More importantly, the P2 effect can also be elicited in an exclusionary scenario: An increase in amplitude was observed if the credibility of the game was increased by the physical presence of the co-players in the lab [19]. Here, the frontal ERP effect was linked to a change in the valence of the relevant event (ball reception), and related to the activation of a reward system [44]. Following this idea, a reduction of the cues provided by a conductor will probably increase the valence of the signal. A corresponding interpretation was recently put forward for a feedback-related P2 [45].

In sum, the first experiment based on the Cyberband setup indicates that a mere reduction of involvement in a social scenario is not sufficient to evoke a strong violation of subjective expectancies and a clear threat to social needs. However, the experimental manipulation might affect the appraisal of social cues.

## Introduction: Experiment 2

The second experiment aimed to replicate the findings from experiment 1, namely the P2 effect. Furthermore, we took advantage of a Cyberband characteristic not shared by Cyberball: ERP analysis will now focus on joint actions which are required if cues are directed to more than one instrument. According to Sebanz and colleagues [20], this expansion of cues might imply that the co-player’s are more actively represented. As indicated by previous studies, this activation is characterized by an attentional allocation to these internal representations which can modulate an exclusionary experience [22,23]. Given that the activation of the aforementioned mechanism affects the self-reported threat, it might also affect the expression of the ERP components related to the processing of exclusionary cues.

Experiment 2 therefore included two experimental conditions referring to joint actions: The cues either required synchronous activity of two instruments (*duet*: e.g., the participant and a co-player), or the synchronous activity of all instruments (*tutti*: the participant and both co-players). Following Sebanz et al. [20], the first manipulation will lead to a phasic activation of the representation of one of the co-players, the second will affect the representation of all co-players. As in experiment 1, the number of the relevant cues will be reduced, whereas the cues exclusively for the co-players will be selectively enhanced.

Based on the result of the first experiment, we stated the following research questions and hypotheses: [1] Is the expression of the P3 effect enhanced by the reduction of joint action as contrasted to the single action? We hypothesized that a more active representation of the co-players [20] in joint-action conditions would enhance the monitoring of the interaction processes. This will increase the sensitivity to exclusionary cues and the adjustment of expectancies on participation – as expressed in the P3 effect [26]. In case that a P3 effect will be observed – which was not significantly expressed in experiment 1 –, we can also assume that an expectancy violation process can be triggered in a hierarchical setting. [2] Can we replicate the frontal P2 effect? If a reduction of cues provided by the superordinated agent (conductor) will affect the salience the signal, the P2 effect is also expected in the joint-action conditions. In contrast to the P3 effect, the P2 effect was not expected to be enhanced in joint as contrasted to single action. [3] Self-reports: As mentioned above, we assume that participants respond more sensitively to a reduced participation in the joint-action scenarios as compared to the single action required in experiment 1. This sensitization will not only affect the expression of the P3, but also the self-reported expression of the threat to social needs. In line with previous findings [22,23], we therefore expected the threat to the need for belonging to be more expressed as compared to experiment 1.

In an exploratory analysis, we also focused on the processing of relevant cues that are not reduced in probability between the sets. The mere descriptive analysis of the grand-averaged ERP responses to the ‘tutti’ cues in experiment 1 (see S1 Figure) suggested that an effect in the P3 range can be observed even if the probability of the events remained unchanged. This ‘indirect’ P3 effect was explored in more detail in experiment 2.

## Methods: Experiment 2

### Participants

In contrast to experiment 1, the current study also included a group comparison: Accordingly, the *a priori* calculation of the sample size was focused on the detection of an interaction between the within-factor “experimental block” (two sets) and the between-factor “group assignment” (three groups). Please note that the data of one experimental group of experiment 1 (*solo*) were also included in the analysis. A corresponding 3x2 design was used in a previous study [11], and reported a mid-sized effect (*f* = 0.2 adjusted to the taxonomy of Cohen, η^2^_p_ = 0.04). According to G*Power [25], a sample size of 66 participants (22 per group) was required to detect an effect with a power of 80% and an alpha at 0.05.

In addition to the 23 data sets (experiment 1: *solo*), a total sample of 52 participants (40 female, 11 male, 1 diverse, age range: 18–38 years) for the two joint-action groups (*duet* and *tutti*) was recruited at the FU Berlin. Most participants were undergraduate students. EEG data were rigorously checked for artefacts (see: Data analysis), leaving a final sample comprising 44 participants. Participants were randomly assigned to the experimental groups. The experimental group ‘duet’ comprised 22 participants (17 female, 4 male, 1 diverse, mean age: 22.32, SD: 3.82), and 16 participants reported playing an instrument. The experimental group ‘tutti’ comprised 22 participants (16 female, 6 male, mean age: 21.00, SD: 4.68), and 12 participants reported playing an instrument. Combined with the 23 participants from experiment 1, the total sample size (n = 67) met the requirements of the power analysis. The effect of ‘expertise’ (playing an instrument) was considered in an a posteriori analysis, which also included the data of study 1. As detailed in the section ‘Limitations’, we found no evidence that the effects of exclusion on self-reports and ERPs were differently expressed in individuals with (n = 38) and without (n = 29) musical expertise.

### Task and design

The instruction and setup (see Fig 1b) was identical to experiment 1. The sequence and timing of a trial was also kept constant (see Fig 2): The symbol of a hand was presented below of the icon representing the conductor. In the EG (experiment group) ‘*duet*’, joint action of two players was indicated by the presentation of two hands, and the index fingers were directed to the position of the instruments to be played (see Fig 3b). The duet cue was relevant if one index finger pointed to the instrument selected to the participant. In the EG ‘*tutti*’, joint action of all players was indicated by the presentation of the palm of a hand (see Fig 3c). In both experimental groups, *solo* cues were also included as a control. For these – less frequent – events, probability was not changed between sets.

Again, a speeded response (time window 750 ms following the onset of the cue) was required if a relevant cue was presented. In feedback period signaled by a visual cue (sheet music, duration: 1.000 ms), participants only received auditory feedback if they responded correctly and in time: Auditory feedback to a solo cue was a sample of the instrument self-assigned to the participant, feedback to a *tutti* cue was a sample including all instruments, and feedback to a *duet* cue was a sample of the participant’s instrument and the one assigned to the corresponding co-players. Following the feedback signal, an inter-stimulus-interval (ISI) varying between 1,000 and 2,000 ms preceded the onset of the next trial. Eye blinks were encouraged within this period.

As in experiment 1, each participant received practice trials (EG *duet*: three duets for each combination, and one solo for each instrument, EG *tutti*: nine tutti and one solo for each instrument). In the EG *duet*, the two sets each comprised 150 cues: In set 1, cues required each 30 duets for the three possible combinations, and 20 soli for each instrument. In set 2, the number of duets including the participants was reduced from 60 to 30, whereas the number of duets of the coplayers remained. As for the *solo* cues, the number remained unchanged for the participant [20], but was increased for each co-player [35]. In the EG *tutti*, each of the two sets comprised 120 cues: In set 1, cues required 60 *tutti*, and 20 *soli* for each instrument. In set 2, the number of tutti cues was reduced from 60 to 30. As in the EG *duet*, the number of soli remained unchanged for the participant [20], but was increased for each co-player [35]. As in experiment 1, the successive sets were interrupted by a short break of approximately three minutes. The experimental design is provided as a table in the supplement (supplement S1 Table).

The analysis of the P2 and P3 effect (as stated in the hypothesis) was focused in the relevant event for which probability was reduced by 50%: In both experimental groups, the corresponding number of trials was 60 in set 1 and 30 in set 2. Additionally, we analysed the response to the relevant cues (*solo*: n = 20 in each set) not changing in probability. This descriptive analysis seeks to identify whether a P3 effect – irrespective of the stable probability of the event.

As in experiment 1, the questionnaires on the need threat (NTQ) and negative mood had to be filled out following set 2. Again, the relative rating requiring a comparison of the sets was required. Finally, participants were debriefed, and written consent was to be confirmed. The completion of the experiment – including the electrode montage – required approximately 90 minutes.

### EEG recording

To allow a statistical comparison of the ERP effects with the data recorded in experiment 1, the same settings for EEG recordings were applied. Also, the offline analysis based on the ‘Vision Analyzer’ software (Version: 2.1, Brain Products, Gilching, Germany) implied the same five consecutive steps as detailed in experiment 1.

In accordance with our previous Cyberball studies, we discarded the data of a participant from analysis if the averaged ERP response to the event of interest (*duet*, respectively *tutti* cue) relied on less than 15 trials. As for *duet* group, the mean number of trials included in the analysis was 44.3 (SD: 10.45), reflecting a trial rejection rate of 26% in set 1. In set 2, the mean number of trials included in the analysis was 21.3 (SD: 4.00), reflecting a trial rejection rate of 29%. As for *tutti* group, the mean number of trials included in the analysis was 46.4 (SD: 7.08), reflecting a trial rejection rate of 23% in set 1. In set 2, the mean number of trials included in the analysis was 22.0 (SD: 4.13), reflecting a trial rejection rate of 27%.

As for the ERP analysis of the cue ‘solo’, only 20 trials were presented in each set. In the *duet* group, in set 1 the mean number of trials included in analysis was 15.0 (SD: 3.01), reflecting a trial rejection rate of 25%. In set 2, the mean number of trials included in the analysis was 15.9 (SD: 2.23), reflecting a trial rejection rate of 21%. As for *tutti* group, the mean number of trials included in the analysis was 16.1 (SD: 2.69), reflecting a trial rejection rate of 19.5% in set 1. In set 2, the mean number of trials included in the analysis was 15.9 (SD: 2.72), reflecting a trial rejection rate of 21%.

### Data analysis

#### Behavioral and questionnaire data.

As in experiment 1, the dependent variables for the self-reports relied on the relative judgement (Δset 2 – set 1). In a first general analysis, the relative ratings of all individuals in experiment 1 and experiment 2 (n = 67) were considered, and for the scales of interest (threat to belonging, negative mood), a one-sided t-test was computed. A subsequent ANOVA included the between-subject factor ‘group’ (*solo* vs *duet* vs *tutti*), considering Greenhouse-Geisser corrected p-values. If a significant group difference was signaled, one-sided t-tests were computed independently for the groups. This procedure was also applied to the remaining NTQ scales (self-esteem, meaningful existence, control). Since no explicit hypothesis was stated for these scales, the analysis was exploratory, and the results will be discussed descriptively.

#### ERP data.

As mentioned above, the GFP index used to determine the time regions of analysis already considered the grand-averaged ERPs of the data sample of experiment 2. Accordingly, the time range of the P3 was defined between 340 and 420 ms, and the time range of the P2 was defined between 200 and 240 ms. For each participant, mean amplitudes were computed separately for the experimental condition ‘set’ and electrodes. For the P2 analysis, peak amplitudes and latencies were additionally determined (see experiment 1).

To test whether the P2 and P3 effect is elicited in ERP responses to the solo cues – not changed in probability between sets – the same time ranges were applied.

As for the P3 segment, a 3 x 2 x 4 ANOVA was conducted comprising the between-subject factor ‘group’ (*solo* vs *duet* vs *tutti* reduction) and the repeated-measure factors ‘set (1 vs 2) and electrode position (Cz, Pz, P7, P8). For the P2 amplitude and latency, a corresponding 3 x 2 x 4 analysis was performed – but including the frontal electrode leads (AFz, Fz, F3, F4). The results will report the Greenhouse-Geisser corrected p-values. In case of a significant interaction, post-hoc comparisons were conducted. SPSS (version 27, IBM) was used for the statistical analysis.

In the same fashion, the ERP responses to the solo cues were analysed. Here, the between-subject factor ‘group’ did only the include the groups EG “*duet” and the EG “tutti”,* resulting in a 2 x 2 4 ANOVA. Moreover, the analysis did also include the responses to the event “*solo* cue for other instrument”. ERPs did not show a systematic difference between the sets (see supplement: S2 Figure).

## Results: Experiment 2

### Self-reports and reaction times

The first step of analysis was focused on the expression of the threat to belonging and negative mood (see Table 1). Independent of group assignment, the reduction of relevant cues increased the threat to belonging, *T(66)=4.88, p < 0.001, d = 0.596*, and negative mood, *T(66)=2.93, p = 0.002, d = 0.358*. Although both effects appear to be more expressed in the group ‘duet reduction’, the ANOVA did not indicate a significant group difference (belonging: *F(2,64)=0.92, p = .405, η*^*2*^_*p*_* = .028*; negative mood: *F(2,64)=0.40, p = .670, η*^*2*^_*p*_* = .012*).

As for the remaining scales (see Table 1), the threat perception was also significantly enhanced when the three groups – including the data of the *EG solo* – were summarized (meaningful Existence: *T(66)=5.10, p < 0.001, d = 0.623*; self-esteem: *T(66)=3.85, p < 0.001, d = 0.470*; control: *T(66)=2.41, p = 0.009, d = 0.294*). Significant differences between the groups were not expressed for the scale ‘meaningful existence’, *F(2,64)=0.748, p = .477, η*^*2*^_*p*_* = .023*, and for the scale ‘self-esteem’; *F(2,64)=1.28, p = .285, η*^*2*^_*p*_* = .038*. However, for the scale ‘control’ a significant group difference was found, *F(2,64)=7.03, p = .002, η*^*2*^_*p*_* = .180*. This effect was due to a higher threat to control in the EG *tutti* as compared to the EG *solo* (experiment 1), *p = .001*, and to the EG duet, *p = .095*. The differences between the groups *solo* and *duet* were negligible, *p = .407*.

A 3x2 ANOVA considering the factors ‘group’ and ‘set’ also indicated that the latencies of response time did not vary significantly between the sets, *F(1, 64)=1.45, p = .233, η*^*2*^_*p*_* = .022*, and the three groups, *F(2, 64)=1.071, p = .349, η*^*2*^_*p*_* = .032*. Moreover, there is no evidence for a significant interaction of the factors, *F(2, 64)=.77, p = .469, η*^*2*^_*p*_* = .023*.

### ERPs: Effects of cue reduction

In the first step on ERPs analysis, we focused on the events (here: cue onset) which differed with respect to frequency between the two sets (‘duets’ in EG duet, and ‘tutti’ in EG tutti). *A corresponding reduction was defined in Cyberball studies as a ‘transition to partial exclusion`.* Fig 3b depicts the effects of the reduction of duet cues and tutti cues. In line with experiment 1, the time course of the grand-averaged ERP is characterized by a frontal positivity (P2) and a sustained positive shift a centro-parietal leads (P3). Descriptive values are provided in Table 2.

The first analysis was related to the P3 effect, and its modulation by the type of action: Involving the data of experiment 1 (*solo*), the P3 components were analyzed running a 3 (group) x 2 (set) x 4 (electrode position) ANOVA. Considering all experimental groups, a significant effect of the factor ‘set’ was found, *F(1, 64)=11.56, p = .001, η*^*2*^_*p*_* = .153*, indicating an increase in amplitude in the second block. However, Fig 3 already indicates that this effect is more expressed in the EG *tutti*, and a corresponding interaction of the factors ‘group’ and ‘set’ was found, *F(2, 64)=5.00, p = .010, η*^*2*^_*p*_* = .135*. This interaction did not depend on electrode position within the electrode cluster as indicated by the non-significant interaction ‘group’ x ‘set’ x’ electrode, *F(6, 192)=1.06, p = .385, η*^*2*^_*p*_* = .032*. The post-hoc-comparisons indicated that the P3 effect in the EG *tutti* was significantly larger as compared to the EG *solo* in experiment 1, *F(1, 43)=6.97, p = .011, η*^*2*^_*p*_* = .140*, and as compared to the EG *duet*, *F(1, 42)=9.09, p = .004, η*^*2*^_*p*_* = .178*. No difference was found between the EG *solo* and EG *duet*, *F(1, 43)=.004, p = .950, η*^*2*^_*p*_* = .000*.

As shown in Fig 3, the frontal P2 effect can be clearly identified in all groups. The ANOVA confirmed a significant increase of amplitude from set 1 to set 2, *F(1, 64)=15.73, p < .001, η*^*2*^_*p*_* = .197*. Although the effect appears to be expressed more in the experiment 1 (solo), not interaction with the factor ‘group’ was observed, *F(2, 64)=0.77, p = .465, η*^*2*^_*p*_* = .024*. Please note that these effects were replicated for the analysis of the P2 peak amplitude.

### ERP: Effects of non-reduced cues

**Fig 4:**
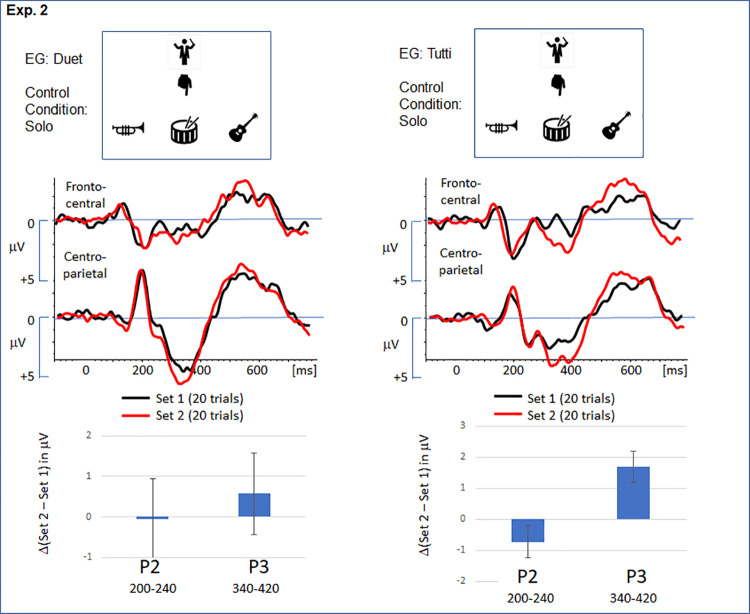
Grand-averaged ERPs for the event “solo cue” not reduced in probability in experiment 2. The ERP traces refer to the response to the relevant cue in set 1 (black) and set 2 (red). The bar graphs represent the mean amplitude of the ERP effect (set 2 – set 1) in the time range of the P2 and the P3. Error bar refer to the SEM. In both experimental groups – duet and tutti – a significant increase of the P3 amplitude in set 2 was observed. The frontal P2 component was not affected.

In a second step on ERPs analysis, we focused on the processing of relevant events (here: cue onset) which did not differ in frequency between the two sets (‘solo cue’ in EG duet and in EG tutti). In case of an event-specific processing, responses were expected to remain stable between the sets. Fig 4 depicts the ERP effects in the two experimental groups for the class of events not reduced in probability between the set (solo cues). As for the reduced event classes (see Fig 3), a frontal positivity (P2) and a sustained positive shift a centro-parietal leads (P3). Descriptive values are provided in Table 3.

**Table 3:** ERPs effects observed for cues not reduced in probability (set 1 vs set 2) in experiment 2.

	Experiment 2 EG Duet		Experiment 2 EG Tutti	
	Set 1	Set 2	Set 1	Set 2
P2 (200–240 ms)	2.45 (.57) 1.30, 3.59	2.38 (.51) 1.36, 3.40	3.00 (.57) 1.86, 4.15	2.08 (.51) 1.06, 3.11
P3 (340–420 ms)	4.36 (.51) 3.33, 5.39	4.93 (.59) 3.74, 6.12	2.17 (.51) 1.11, 3.16	3.81 (.59) 2.62, 5.00

*Caption: For each, the P2 and the P3, the mean amplitudes in the corresponding time ranges (200–240ms, 340–420ms) are provided. In each cell, the first line refers to the mean value and the standard error of mean, and the second line to the lower and upper limits of the confidence interval (95%).*

As for the frontal P2, the amplitude was comparable between the sets. This observation is in line with the effect of set provided by the ANOVA, *F(1, 42)=1.60, p = .213, η*^*2*^_*p*_* = .037*. Moreover, the lack of an interaction indicated that a P2 effect was not selectively expressed in one of the experimental groups. *F(2, 42)=1.21, p = .279, η*^*2*^_*p*_* = .028*.

In contrast, the P3 was clearly enhanced in set 2, resulting in a significant main effect of the factor ‘set’, *F(1, 42)=11.23, p = .002, η*^*2*^_*p*_* = .211*. Although the increase appears to be more pronounced in the EG ‘tutti’, the ANOVA did not signal a significant interaction, *F(1, 42)=2.74, p = .105, η*^*2*^_*p*_* = .061*.

## Discussion: Experiment 2

The findings of experiment 2 can be summarized as follows: ERP responses to cues are characterized by a frontal P2 and a centroparietal P3. Reducing the probability of a cue for joint actions enhances the P2 amplitude. A comparable enhancement for the P3 amplitude was primarily expressed if cues required joint actions of all instruments (*tutti*). The reduction of the cues for joint action also affects the processing of ‘solo’ cues: Although the frequency was not changed between sets, an increase in the P3 in set 2 was observed. The analysis of self-reports confirmed that the reduction of cues directed to the participant elicited a small, but reliable threat to belonging. This effect did not differ significantly between the groups. This pattern of results will be discussed in the following.

Following our first hypotheses, the P3 effect induced by cue reduction was expected to be more expressed as compared to experiment 1 (*solo*). As for the EG *duet*, the P3 effect was barely expressed (Δ0.2 μV) and did not differ from the expression of the P3 effect observed for the reduction of solo cues. However, in the EG *tutti*, the reduction of cues elicited a significant increase of the P3 effect (Δ1.6 μV), and the effect size (η^2^_p_ = .520) approached the effect size observed in the same time range in the Cyberball study (η^2^_p_ = .557, [11]). *First, the expression of the P3 effect supports the idea that an expectancy violation process can be activated in a hierarchical setting. Second, the findings partially support the idea* that the activation of the representation of the co-players action sensitizes the participant for a violation of the expected involvement [22,23]: Actions which involve all co-players (*tutti*) are apparently more-closely monitored, which supports the establishment of situation-specific expectations [39]. The increase of the P3 effect signals that a higher sensitivity in the detection of a deviance between the expected and experienced participation [16], and that a corresponding adaptation is initiated [15,35]. In sum, the processing of exclusionary signals in Cyberband approaches the mechanisms elicited in Cyberball if an active representation of the co-players is triggered.

Although experiment 2 indicates that a P3 effect can be elicited in the Cyberband setup if joint action is required, we have no evidence that this manipulation also affects the self-reported threat to belonging or negative mood. In other words, the mere processing of the reduction of self-relevant cues – as signaled by the P2 and P3 effect – is not sufficient to induce an aversive state in the participant. Although previous Cyberball studies already suggested that the degree of expectancy violation – as reflected in the P3 effect – does not necessarily predict the post-hoc evaluation of the participant [26], the dissociation observed in the Cyberband setup is surprising. It suggests that exclusionary cues by a superordinate agent are processed differently from exclusion by equals in a community.

The different processing of the exclusionary cues might be related to the P2 effect, which is reliably observed in both experiments. As mentioned before, the frontal P2 effect might indicate activation of attentional resources and the detection of salient events [41,42]. In the context of the cue distribution provided by the conductor, the increase induced by the reduction might indicate an increase in reward value. This interpretation is in line with the idea that the P2 indicates the processing of (more-)rewarding stimuli affecting the motivational state [46], and reflects the valence of feedback signals [45,47]. Following this interpretation, the frontal P2 effect might be related to the lack of an aversive response, as reflected in the self-reports. If infrequent cues provided by a superordinate agent are more salient and more rewarding, they might buffer the aversive response to the violation of the subjective expectancy.

Other studies, however, indicated that the P2 effect might reflect social comparison processes, and was found to be enhanced in the case of a – subjectively – disadvantageous outcome [48]. This process relates to an early stage of outcome evaluation; therefore, detailed information is not accessible, and processing is relatively rough [49]. Following this interpretation, the P2 effect will serve as an index of the detection of a social deviance and will replace the P3 effect as an (electrophysiological) index of exclusion. The latter might be (less) expressed because the hierarchical setting might prevent an a priori expectation of social involvement.

Finally, experiment 2 confirmed that classes of self-relevant events are not processed independently: The reduction of the ‘duet’ (EG duet), respectively ‘tutti’ (EG tutti) cues did also affect the processing of the solo cues in both groups, and the clear P3 effect serves as an ‘indirect’ marker of the change in probability (Fig 4). To the best of our knowledge, a comparable transfer effect of probability between different classes of events has not been previously described. The P3 effect for the ‘solo’ cues in experiment 2 cannot be classified as a novelty P3 [50] because this class of cue is already introduced in set 1. We like to offer two potential explanations: First, the P3 effect also reflects a comparison process, and is triggered by the (relative) increase of solo cues for co-players in set 2. However, according to previous results, a decrease in the P3 would have been expected [51]. Second, the P3 effect is not event-specific, and responds to the overall-reduction of self-relevant events in set 2. In this case, however, a clear P3 effect is expected for the reduced events (‘duets’ in EG duet, respectively ‘tutti’ in EG tutti), too. In sum, the cognitive mechanism inducing the ‘indirect’ P3 effect (see Fig. 4) remains to be explained in further studies.

## General discussion

In two experiments, we explored the processing of exclusionary signals in a new experimental paradigm, labeled ‘Cyberband’. In contrast to the established Cyberball paradigm, exclusion in the Cyberband can be attributed to the decisions of a superordinate actor (conductor). Moreover, the new paradigm allows us to explore whether exclusionary signals related to single or joint actions are processed differently. Both features affect the processing of a reduction in social involvement.

The differences are summarized in Fig 1: The most-reliable ERP signature for the processing of exclusionary cues in the Cyberball experiment is the P3 effect [13]. It has been related to the detection of deviances between the expected and the experienced participation [14], and to the adjustment of the subjective expectancies [16]. Moreover, the exclusion in Cyberball reliably induces a threat to the need for belonging and enhances negative mood [12]. These markers are reduced in the Cyberband setup suggesting that a mere reduction of self-relevant signals in a (simulated) social setting is not sufficient to elicit reliable physiological and psychological responses to exclusionary social signals. As mentioned above, attribution of exclusion to the decision of a single actor (here: conductor) affects the responses. This idea is in line with earlier findings suggesting that targets of ostracism suffer a greater threat to belonging if a specific cause for their treatment is not provided [52]. Moreover, the reduction of the P3 effect reveals that the strict hierarchy provided in the Cyberband paradigm prevents an individual from establishing a reliable *a priori* expectation of the involvement in social interaction.

The ‘typical’ signatures of exclusion processing – as revealed by Cyberball studies – can rather be observed if participants are excluded from actions involving all co-players (*tutti*). The enhanced sensitivity – as reflected in the P3 effect and in the enhanced threat to control – might be due to a more-active representation of co-players in case of joint actions [20]. Strengthening this representation might approach the internal state induced in the Cyberball experiment.

In contrast to the P3 effect, the frontal P2 effect serves as the most reliable signature for a reduced involvement in the Cyberband setup. The modulation of the frontal positivity (P2) is not established in Cyberball research [13]. In Cyberband, the effect is expressed independently of the type of action (single vs joint), and exclusively in the class of events with reduced probability (see Fig 4). As mentioned above, the P2 effect indicates that rare cues in set 2 are more salient and that an early social comparison process detected a deviance [49]. Notably, this process has not been reported consistently in Cyberball studies. Alternatively, the P2 effect might signal that infrequent cues provided by a superordinate agent are more rewarding, and will therefore modulate the affective and motivational state of an individual [53]. The experimental setup used in the two studies does not allow us to differentiate between the two accounts: Further studies varying the frequency of relevant cues (e.g., preferential treatment of the participant), or the steepness of the hierarchy are therefore necessary to approach the functional significance of the P2 effect in the Cyberband paradigm.

### Limitations and future research

Although the Cyberband paradigm appears to be a promising new approach in the research on social exclusion, its value will have to be validated in further studies. Follow-up studies will have to focus on the noticeable differences between the Cyberband and Cyberball setup. First, the Cyberband setup is more complex, and the number of different events (here: number of possible cues) might complicate the monitoring of frequencies. Second, in Cyberband it is less clear that co-players profit from the exclusion of the individuals. Finally, we have to consider the superior spatial position of the conductor: This position signals a higher social power [54], and this gradient can affect the processing of exclusionary signals [15].

Other limitations are related to our sample: First, differences in the expertise (e.g., membership in an orchestra) might affect expectations on the involvement. In a first analysis, the compared the response to exclusion in Cyberband in individuals with (n = 38) and without (n = 29) musical experience. The threat to exclusion was slightly enhanced in the latter group (p = .070), as well as the expression of the P2 effect (p = 0.90), but we found no evidence for a difference in the expression of the P3 effect (p = .815). However, we cannot completely rule out that familiarity with the scenario affects the processing of the exclusionary signals, Second, our sample is homogeneous concerning the social and educational background of participants. This restricts the generalizability of our results [55].

Another limitation is due to the spatial resolution of the electrophysiological signals: A more fine-graded analysis of the components of interest (P2 and P3) would benefit from an increase in the number of active electrodes, allowing a topographical mapping. This information might support the interpretation of the ERP effects’ functional significance.

Finally, the ‘indirect’ P3 effect observed for non-reduced cues remains to be explained (Fig 4). The experimental manipulation in the two experiments did not allow us to conclude whether the ERP effect signals that different events (e.g., solo cue vs tutti cue) are not sufficiently separated on a higher processing stage. Follow-up studies are required to explore the sensitivity of the system to changes in the probability of different self-relevant events.

## Conclusions

Electrophysiological and self-report data provided converging evidence that the processing of social exclusion is significantly modified in the Cyberband setup. Extending the range of the established Cyberball paradigm, the new paradigm allows us to explore the mechanisms and effects of ostracism in social hierarchies. In contrast to the established Cyberball paradigm, the new setup emphasized the role of social comparison processes, which do not necessarily affect the affective state. The additional consideration of joint actions is an important aspect in social participation and might inform research in educational settings or work organizations.

## Supporting information

S1 Table Experimental Design. Number of trials for the different events in the two experiments. Bold numbers refer to the critical comparison (effect of reduced cue frequency). Part = Participant, CP1 = Co-Player 1, CP2 = Co-Player 2.
(PDF)

S2 Table ERPs effects in experiment 1 and 2. The P2 data are separated for the peak of the amplitude (in uV) and the corresponding latency (in ms). In each cell, the first line refers to the mean value and the standard error of mean, and the second line to the lower and upper limits of the confidence interval (95%).
(PDF)

S1 Fig Grand-averaged ERPs for events of same frequency in the blocks in experiment 1. In block 2, the *tutti* cue triggered a centroparietal P3 in the tome range 360–440ms. The expression of the effect (Δ: 2.5 μV) appears to be stronger expressed in contrast to the ERP response to the cue ‘solo’ discussed above (Δ: 0.8 μV). Since the ERPs are based on a small number of trials, we renounced a statistical comparison. Notably, a frontal P2 effect is not expressed.
(TIF)

S2 Fig Grand-averaged ERPs responses to the cue for co-players (solo for other instrument) in set 1 and set 2 (A) Effects in experiment 1. Despite of the increase in cue frequency, no differences between the sets were observed in the range of the frontal P2, or in the centroparietal P3 range. (B) Effects in experiment 2: Neither in the EG *duet* nor in the EG *tutti*, a modulation of the ERP components P2 and/or P3 was observed between the sets.
(TIF)
