# Supplementary material for: Processing of social exclusion in a strict hierarchy
Source: PLoS One. 2025 Dec 19;20(12):e0338212. doi: 10.1371/journal.pone.0338212 (PMC12716733; doi:10.1371/journal.pone.0338212)
Supplement: S1 Table — Number of trials for the different events in the two experiments. Bold numbers refer to the critical comparison (effect of reduced cue frequency). Part = Participant, CP1 = Co-Player 1, CP2 = Co-Player 2. (PDF) [file pone.0338212.s001.pdf]

S1 Table

		Experiment 1<br><br>Solo Reduction		Experiment 2<br><br>Duet Reduction		Experiment 2<br><br>Tutti Reduction	
		Set 1	Set 2	Set 1	Set 2	Set 1	Set 2
Solo	Part	**40**	**20**	20	20	20	20
	CP1	40	50	20	35	20	35
	CP2	40	50	20	35	20	35
Duet	Part – CP1	-	-	**30**	**15**	-	-
	Part – CP2	-	-	**30**	**15**	-	-
	CP1 – CP2	-	-	30	30	-	-
Tutti	ALL	10	10	-	-	**60**	**30**

S1 Table: Experimental Design. Number of trials for the different events in the two experiments. Bold numbers refer to the critical comparison (effect of reduced cue frequency). Part=Participant, CP1 = Co-Player 1, CP2 = Co-Player 2
