# Supplementary material for: Processing of social exclusion in a strict hierarchy
Source: PLoS One. 2025 Dec 19;20(12):e0338212. doi: 10.1371/journal.pone.0338212 (PMC12716733; doi:10.1371/journal.pone.0338212)
Supplement: S2 Table — The P2 data are separated for the peak of the amplitude (in uV) and the corresponding latency (in ms). In each cell, the first line refers to the mean value and the standard error of mean, and the second line to the lower and upper limits of the confidence interval (95%). (PDF) [file pone.0338212.s002.pdf]

S2 Table

Results of the peak analysis of the P2

	Experiment 1<br>Solo		Experiment 2<br>EG Duet		Experiment 2<br>EG Tutti	
	Set 1	Set 2	Set 1	Set 2	Set 1	Set 2
P2 peak	4.37 (.37)<br>3.60, 5.15	6.11 (.51)<br>5.05, 7.18	3.85 (.37)<br>3.12, 4.59	4.75 (.48)<br>3.78, 5.72	4.62 (.37)<br>3.90, 5.37	5.38 (.48)<br>4.41, 6.34
P2 latency	204.8 (4.1)<br>195.4,<br>213.9	209.5 (4.5)<br>200.1,<br>218.8	195.8 (4.5)<br>186,8,<br>204,8	198.5 (4.4)<br>189.7,<br>207.4	193.0 (4.5)<br>184.0,<br>202.0	193.2 (4.4)<br>184.3,<br>202.0

S2 Table: ERPs effects in experiment 1 and 2. The P2 data are separated for the peak of the amplitude (in uV) and the corresponding latency (in ms). In each cell, the first line refers to the mean value and the standard error of mean, and the second line to the lower and upper limits of the confidence interval (95%).

Statistical effects: Results of a 3 x 2 x 4 ANOVA (group x set x electrode)

The frontal P2 effect can be clearly identified in all groups. The ANOVA confirmed a significant increase of amplitude from set 1 to set 2, $F(1, 64)=22.75$, $p<.001$, $\eta^2_p =.262$. This increase did not vary significantly between the groups, $F(2, 64)=1.75$, $p=.182$, $\eta^2_p =.052$. As for the P2 latency, no significant differences were obtained between the sets, $F(1, 64)=1.20$, $p=.277$, $\eta^2_p =.018$, a corresponding difference can also not be found in single groups as indicated by the interaction ‘set’ and ‘group’, $F(2, 64)=.33$, $p=.720$, $\eta^2_p =.010$.
