# Supplementary figures and images for: Processing of social exclusion in a strict hierarchy
Source: PLoS One. 2025 Dec 19;20(12):e0338212. doi: 10.1371/journal.pone.0338212 (PMC12716733; doi:10.1371/journal.pone.0338212)

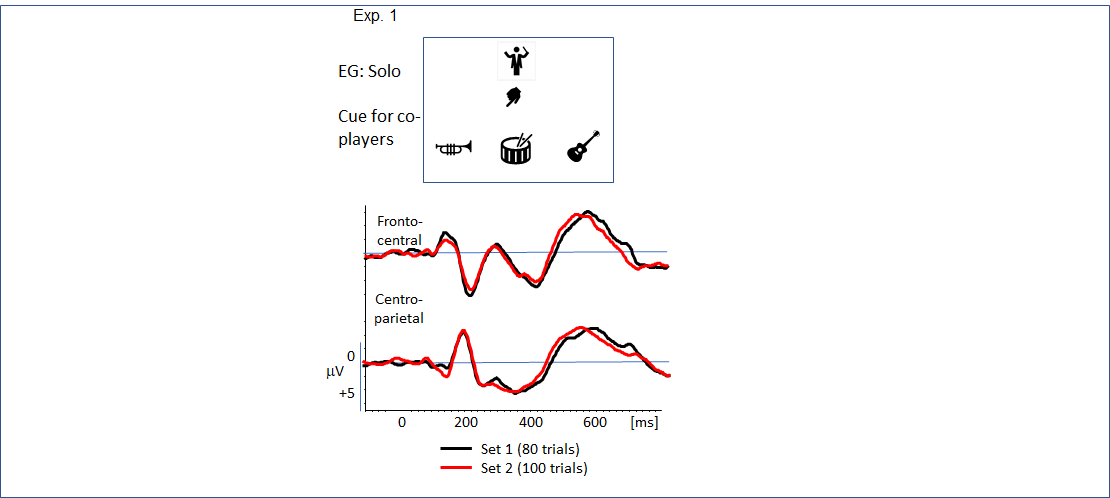

Supplement: S1 Fig — In block 2, the tutti cue triggered a centroparietal P3 in the tome range 360–440ms. The expression of the effect (Δ: 2.5 μV) appears to be stronger expressed in contrast to the ERP response to the cue ‘solo’ discussed above (Δ: 0.8 μV). Since the ERPs are based on a small number of trials, we renounced a statistical comparison. Notably, a frontal P2 effect is not expressed. (TIF) [file pone.0338212.s003.tif]

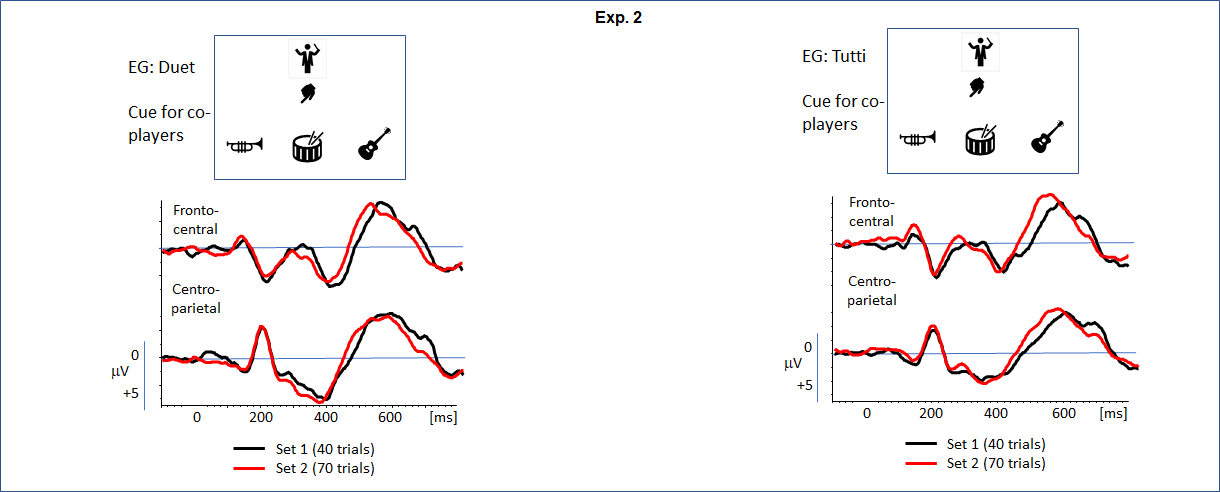

Supplement: S2 Fig — Despite of the increase in cue frequency, no differences between the sets were observed in the range of the frontal P2, or in the centroparietal P3 range. (B) Effects in experiment 2: Neither in the EG duet nor in the EG tutti, a modulation of the ERP components P2 and/or P3 was observed between the sets. (TIF) [file pone.0338212.s004.tif]
